# Facile synthesis of elastin nanogels encapsulated decursin for castrated resistance prostate cancer therapy

**DOI:** 10.1038/s41598-024-65999-x

**Published:** 2024-07-02

**Authors:** Gulzar Ahmed Rather, Preethi Selvakumar, K. Satish Srinivas, K. Natarajan, Ajeet Kaushik, Prabhakar Rajan, Seung-Rock Lee, Wong Ling Sing, Mohammad Alkhamees, Sen Lian, Merrel Holley, Young Do Jung, Vinoth-Kumar Lakshmanan

**Affiliations:** 1 Prostate Cancer Biomarker Laboratory, Faculty of Clinical Research, Sri Ramachandra Institute of Higher Education & Research, Porur, Chennai, 600116 India; 2 Department of Radiation Oncology, Faculty of Medicine, Sri Ramachandra Institute of Higher Education & Research, Porur, Chennai, 600 116 India; 3 Department of Urology, Faculty of Medicine, Sri Ramachandra Institute of Higher Education & Research, Porur, Chennai, 600 116 India; 4 NanoBioTech Laboratory, Department of Environmental Engineering, Florida Polytechnic University, Lakeland, FL USA; 5 Centre for Cancer Cell and Molecular Biology, Cancer Research, Barts Cancer Institute, UK City of London Centre, Charterhouse Square, London, EC1M 6BQ UK; 6 Department of Biochemistry, Chonnam National University Medical School, Seoyang Ro 264, Hwasun, 58128 Korea; 7 Faculty of Health and Life Sciences, INTI International University, Persiaran Perdana BBN Putra Nilai, 71800 Nilai, Negeri Sembilan Malaysia; 8 Department of Urology, College of Medicine, Majmaah University, 11952 Al Majmaah, Saudi Arabia; 9 Department of Biochemistry and Molecular Biology, School of Basic Medical Sciences, Southern Medical University, Guangzhou, Guangdong China; 10 International Hyperbaric Medical Foundation, The Tissue & Organ Regeneration Institute, Greater New Orleans, USA

**Keywords:** Elastin, Nanogel, Decursin, Drug release, Prostate cancer therapy, Nanoparticles, Prostate

## Abstract

Nanogels offer hope for precise drug delivery, while addressing drug delivery hurdles is vital for effective prostate cancer (PCa) management. We developed an injectable elastin nanogels (ENG) for efficient drug delivery system to overcome castration-resistant prostate cancer (CRPC) by delivering Decursin, a small molecule inhibitor that blocks Wnt/βcatenin pathways for PCa. The ENG exhibited favourable characteristics such as biocompatibility, flexibility, and low toxicity. In this study, size, shape, surface charge, chemical composition, thermal stability, and other properties of ENG were used to confirm the successful synthesis and incorporation of Decursin (DEC) into elastin nanogels (ENG) for prostate cancer therapy. In vitro studies demonstrated sustained release of DEC from the ENG over 120 h, with a pH-dependent release pattern. DU145 cell line induces moderate cytotoxicity of DEC-ENG indicates that nanomedicine has an impact on cell viability and helps strike a balance between therapeutics efficacy and safety while the EPR effect enables targeted drug delivery to prostate tumor sites compared to free DEC. Morphological analysis further supported the effectiveness of DEC-ENG in inducing cell death. Overall, these findings highlight the promising role of ENG-encapsulated decursin as a targeted drug delivery system for CRPC.

## Introduction

Prostate cancer (PCa) is frequently identified as the most prevalent type of cancer among males. Despite its typically slow-progressing nature, PCa continues to be the third-highest contributor to male cancer-related mortality^1^. Despite recent advances in detection and localized curative treatment for prostate cancer, 23- 40% of those patients will go on to develop metastatic disease. Castration-Resistant Prostate Cancer (CRPC) arises when hormone –refractory growth occurs in a castrate androgen level environment. New therapeutic approaches involving the use of natural products to promote tumor regression and apoptosis and pause primary and secondary metastasis and angiogenesis have been widely studied. Interestingly, research has indicated that phytochemicals can selectively affect the signaling of the androgen receptor (AR) and target PCa stem cells (PCSCs)^2^. However, their therapeutic efficacy in preclinical and clinical trials has been undermined by their unfavorable pharmacological properties such as their hydrophobicity, instability, low pharmacokinetic profile, high excretion rate, and poor water solubility^3^. Based on testosterone levels and available treatments, PCa can be categorized as androgen sensitive or androgen insensitive. AR antagonists or a combination androgen blockade strategy have emerged as an appealing and promising option for PCa therapy. Both non-steroidal medications like flutamide, bexarotene, and enzalutamide and steroidal medications like cyproterone acetate are among the most often recommended AR antagonists for PCa. Current treatment options available for PCa are radiation therapy, chemotherapy, hormonal therapy and surgery. But nearly all current treatments is associated with the severe side effects like toxicity, fatigue, hair loss and peripheral neuropathy and therefore, it is crucial to explore innovative and new treatment options that are not only cost-effective but also minimize or eliminate side-effects and enhance the overall effectiveness^4,5^. Nanodrug delivery systems are becoming a potential therapeutic approach for treating patients with prostate cancer (PCa). They offer the potential to enhance clinical outcomes by improving drug delivery to the affected site, increasing drug effectiveness, and minimizing adverse effects on healthy tissues^6^.

Nanogels are a distinctive category of polymeric nanomaterials employed in drug delivery systems, designed to encapsulate both hydrophobic and hydrophilic drugs^7^. Nanogels are 10–200 nm-sized hydrogels made from three-dimensional networks that are physically or chemically crosslinked^8^. They are ideal for use as a drug delivery vehicle owing to their remarkable softness, drug-loading capacity, porosity, bioavailability, and biocompatibility^9^. Due to their tendency to undergo swelling, nanogels exhibit significant drug-loading capacity and encapsulation efficiencies. They can encapsulate more drugs than other nano-carriers and promote the sustained release of entrapped molecules. Nanogel, a polymeric material, provides a platform for encapsulating biological molecules, therapeutics, and even nanoparticles^10^. To address the challenges of cancer treatment, a wide range of drug delivery nanoplatforms have been developed, including liposomes, polymer micelles, nanoparticles, and mesoporous silica nanoparticles. However, in these carriers, drug loading is achieved by physical adsorption resulting in immediate release by diffusion after administration^11–13^. The development of multiresponsive nanogels which respond to different stimuli has advanced significantly in recent years, with the goal of improving the efficacy of drug delivery systems^14^. These developments include the synthesis of cytocompatible and biodegradable nanogels. Among the various external stimuli like temperature, pH and light irradiation, pH has shown more interest, this is because various tissues and organelles, such as endosomes/lysosomes, extra-tumoral environments, and inflamed regions, experience a substantial decrease in pH than normal tissues^15^. Therefore, development of pH-responsive nanocarriers seems like a good way to improve the therapeutic usefulness of nanocarrier-based drug delivery systems.Recenty periodic mesoporous organosilica nanoparticles (nano PMOS), Mesoporous Silica and Oligo Methacrylates-Based Dual-Responsive Hybrid Nanogels were used for targeted chemotherapy of Prostate cancer^16,17^.However, there is limited literature on pH-responsive nanogels loaded with anticancer drugs specifically for prostate cancer therapy.

Elastin is well known for its biocompatibility, inertness, and biodegradability. It is also mechanically tunable and responsive to pH and temperature changes. Furthermore, it can easily cross the blood–brain barrier^18,19^. Moreover, the growing interest in elastin as a biomaterial is due to its properties of elasticity, self-assembly, long-term stability, and biological activity. Notably, the attributes of elasticity and biological activity of elastin in combination with natural or synthetic polymers are utilized to model and improve the mechanical properties of biomedical products and drug delivery systems^20^.

Natural compounds like tocotrienols, sulforaphene, ginsenosides, ursolic acid, berberine, honokiol, xanthohumol, oridonin, curcumin, kaempferol, genistein, tannic acid, epigallocatechin-3-(EGCG), and resveratrol have demonstrated remarkable potential as effective agents against PCa in vitro and in vivo studies, suggesting their potential for use as anti-PCa treatments^2^.The coumarin compound decursin (DEC), isolated from *Angelica gigas Nakai* (AGN), was explored in our laboratory. DEC is well known for its anti-inflammatory, anticancer, antibacterial, antifungal, and anti-osteoclastic activities^11^. In both extract and nano-formulations, DEC and its isomer decursinol angelate have demonstrated enhanced wound healing activity^21,22^.Our group recently reported the preparation of elastin nanogels^12^. However, the novelty of this study lies in the fact that DEC has been first time explored in an elastin -based nanocarrier for prostate cancer therapy, marking a significant advancement in the field.

In this study Elastin-based nanogel ENG was prepared by inverse mini-emulsion technique in which elastin was crosslinked with bis (sulfosuccinimidyl) suberate (BS3) via amide bond formation and characterized by different techniques. Following that, the elastin nanogel (ENG) was loaded with a model drug (decursin, denoted as DEC), for the very first time. Comprehensive analyses were performed to evaluate critical parameters such as drug loading efficacy, encapsulation efficiency, drug release profile, cytotoxicity, and morphological properties.

## Results and discussions

### Preparation/Particle size distribution/Zeta potential/Polydispersity

**Figure 1:**
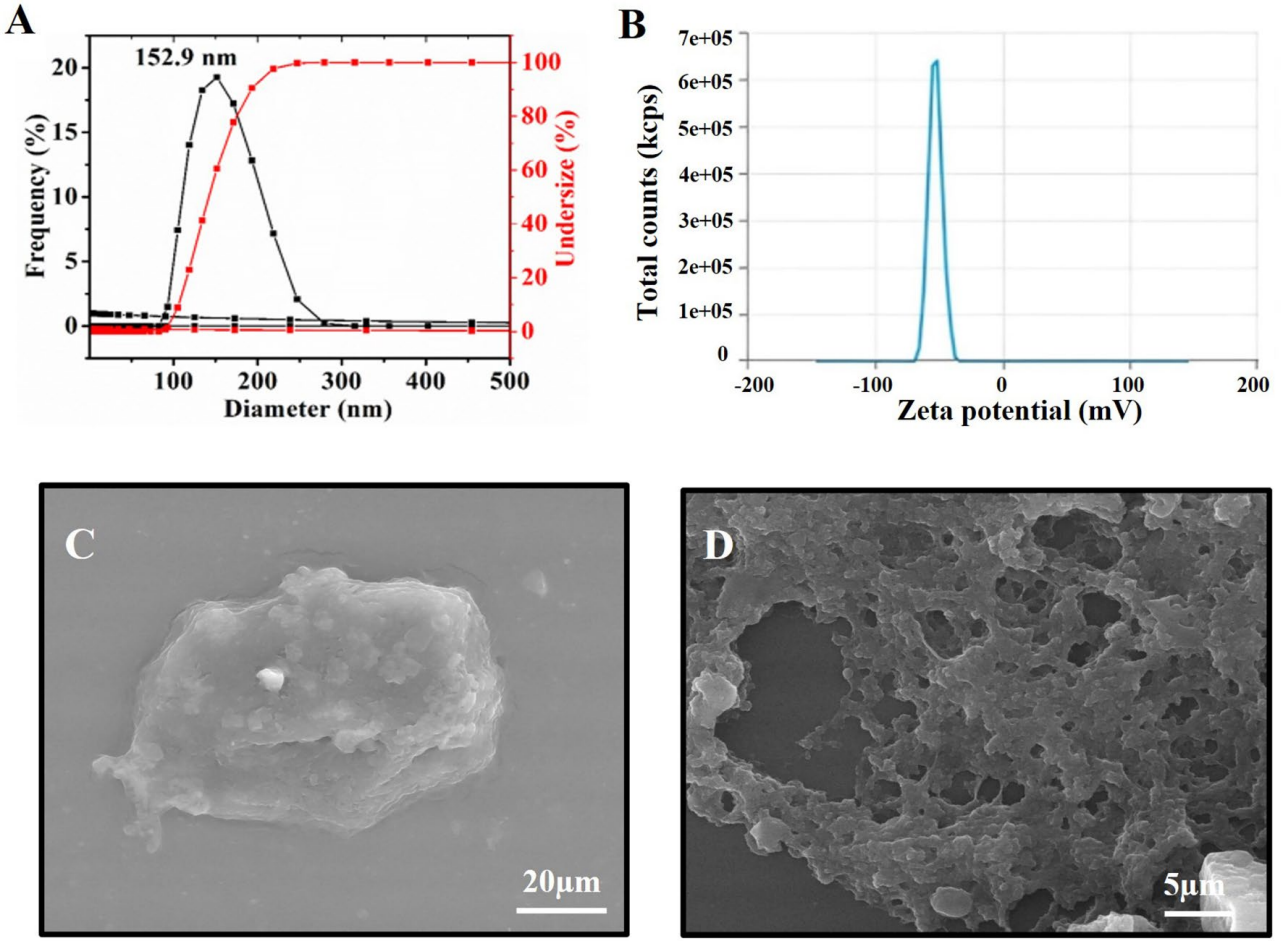
(**A**) shows the particle size distribution of ENG by DLS analysis, (**B**) Zeta potential, (**C**) and (**D**) refers to SEM micrographs of ENG and ENG-loaded DEC.

In present study preparation and characterization of ENG was carried out using inverse-mini emulsion technique .BS3 was used as a cross-linker that binds to elastin via the amino group of lysine through a nucleophilic substitution reaction to form an amide bond. The terminal sulpho-NHS group separates from the BS3, facilitating the formation of an amide bond with the lysine group present in elastin^23,24^. The particle size distribution of ENG were determined using DLS .The ENG was diluted in water and the average size of the ENG was was found to be 152.9 nm (Fig. 1A). The ZP of ENG was analyzed in water at 25 °C. ENG showed a ZP of − 52.67 ± 1.6 mV (Fig. 1B). The high negative value of zeta ptential indicated the high stability of the particles; thus, no external stabilizing agent was needed^5^. The PDI value of the designed ENG was found to be 0.334 (Table 1), which indicated that the particles were homogeneous. The lower the PDI value, the higher the uniformity of particles in the sample ^25^.

**Table 1:** Physicochemical characterization of ENG.

Sample	Particle size (nm)	Zeta potential	Polydispersity index	Encapsualtion efficiency (%)
ENG	152.9 ± 0.968	− 52.67 ± 1.6	0.334	91.14

### Morphological analysis of ENG

The SEM analysis showed that the ENG was spherical and spongy in shape with an average particle size of 88.95 nm (Fig. 1C). The variation in the particle size observed using DLS and SEM analyses is due to the difference in the sample preparation for the two analyses. The SEM analysis was performed using dried samples, whereas the DLS analysis was conducted using a solution and, thus, the particles were swollen in water^26^. The DEC –loaded ENG as shown in (Fig. 1D), shows variation in morphology after the addition of drug. The drug-loaded nanogel exhibits a spherical, porous morphology with a size of 410 nm. It is evident that the size of the DEC-loaded nanogel differs from that of the nanogel alone, primarily due to the aggregation induced by DEC loading. Consequently, the morphology of DEC-loaded nanogels exhibits variation compared to unloaded nanogels. Moreover, the agglomeration effect of nanogels in solid state influences the evaluation of particle size by SEM^31^.

### Thermo-gravimetric (TGA) analysis

**Figure 2:**
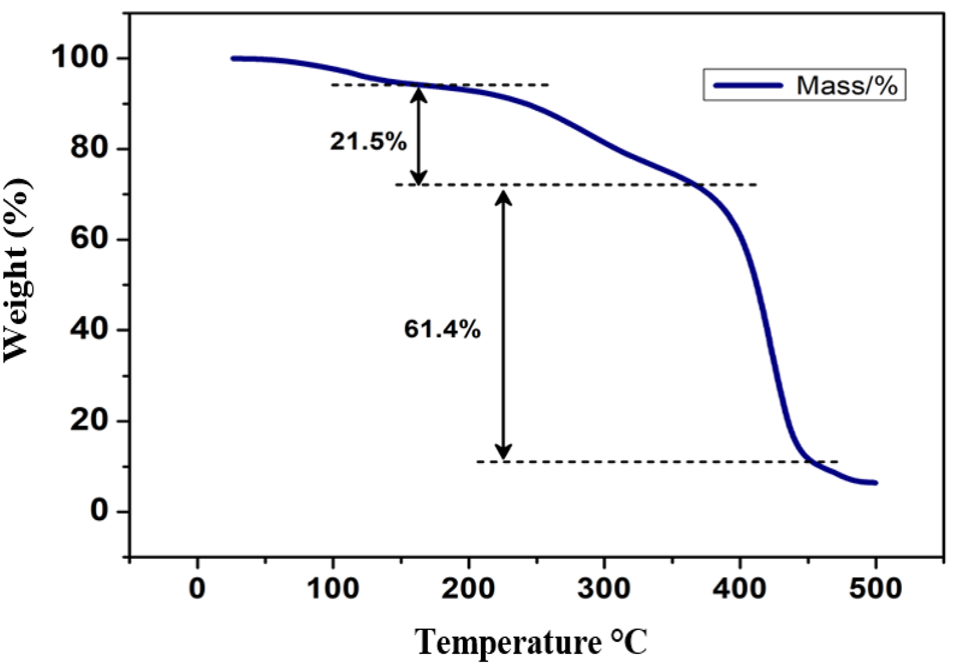
Thermo-gravimetric analysis of the ENG.

The thermal stability of ENG was assessed using TGA **(**Fig. 2**).** The TGA graph showed that decomposition occurred at around 100 °C due to the moisture and other solvents in the sample. A second decomposition, with a weight loss of 21.5%, occurred at approximately 207 °C; this was probably due to the presence of starting materials because of partial decomposition. The next decomposition occurred at 364 °C, with a loss of 61.4% and leftover residue of 6.41%, which indicated the complete decomposition of the compound. Based on the differential thermal analysis (DTA), a strong endothermic peak was observed at 429 °C, probably due to the loss of structure in parallel with the transformation to a crystalline state.

### Swelling study

**Figure 3:**
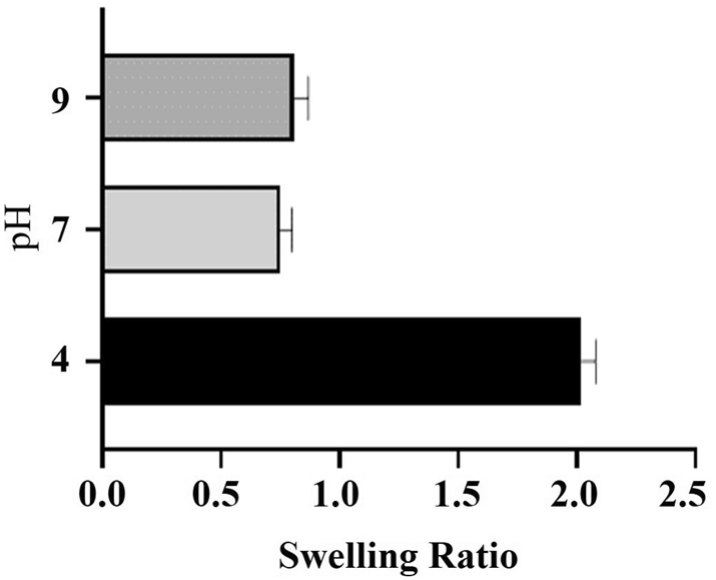
Swelling study of ENG at three different pH ranges. Data are presented as the mean ± SD (n = 3).

The swelling of the ENG was studied at three different pH ranges—acidic, neutral, and basic (Fig. 3). It was found that the ENG swelled more in acidic conditions than in neutral and alkaline conditions. Due to the polyelectrolyte properties of elastin, it swells below its pKa ^9^. This forms the basis of the pH responsivity of ENG. The presence of a crosslinking agent is the key factor that determines the swelling behaviour of nanogels^26^. Moreover, at lower pH, the tertiary amines become protonated and the polymers become more soluble in water. Polymers that show responsivity towards change in pH are capable of delivering therapeutics into the tumoral microenvironment^27^.

### Encapsulation efficiency (EE) and drug loading (DL)

**Figure 4:**
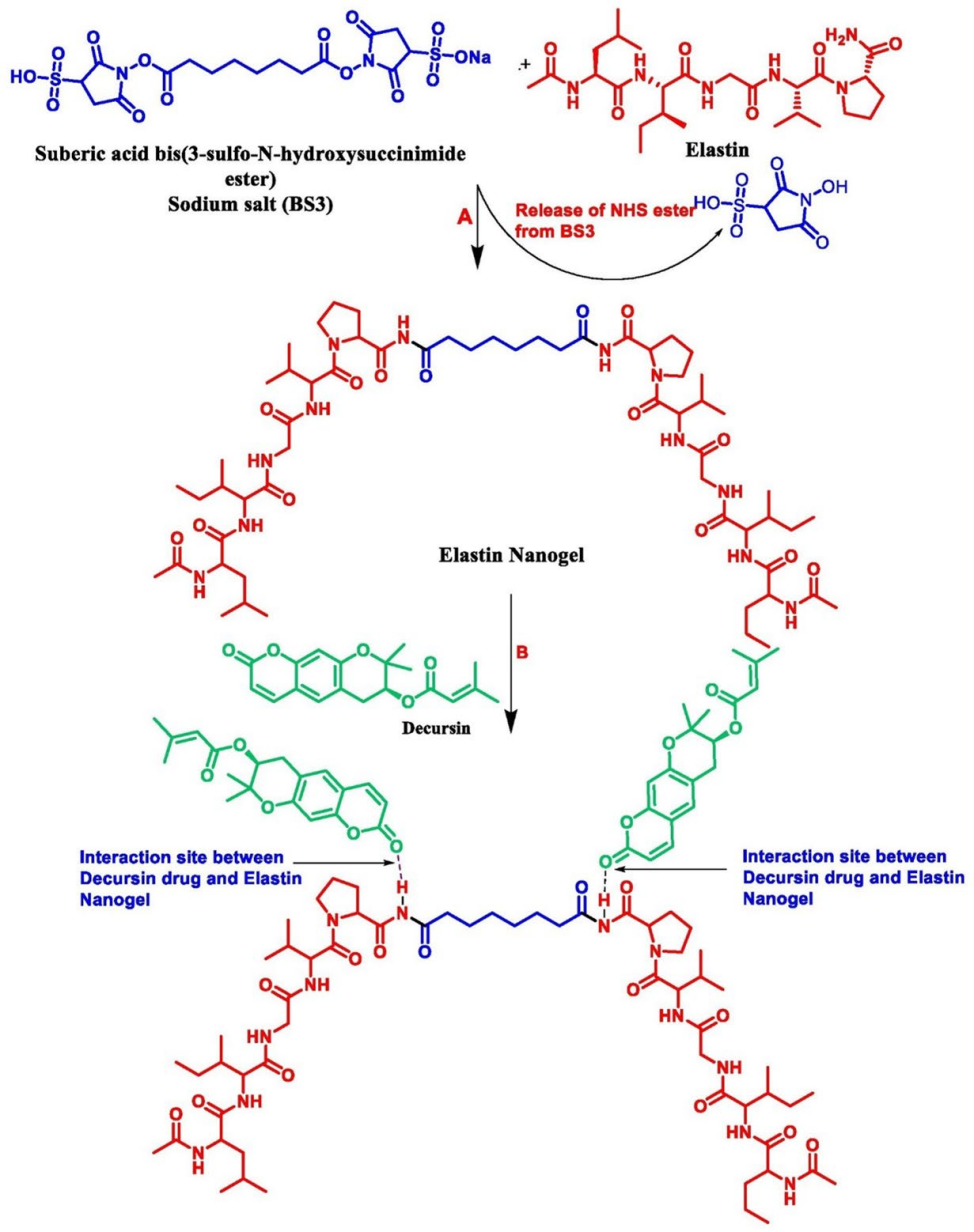
Shows the possible mechanism: At step (**A**) there occurs a reaction between Elastin and BS3 results in the formation of Elastin nanogel by the release of NHS ester in which BS3 acting as crosslinker between two Elastin moiety. At step (**B**) when Decursin is incorporated there is the possible Hydrogen bonding interaction of Decursin with the Elastin Nanogel at a particular interaction site as shown above.

In the experimental studies, the λmax of DEC was found at 330 nm, which is the actual peak of DEC. The calibration curve of DEC was plotted based on the λmax.A linear relationship was observed between the absorbance and DEC concentration from (0–13 µg/mL); the regression equation of the calibration curve was y = 0.2365x-0.0213. From the working standard solution, 3 µL of sample and blank were used to check the maximum absorption peak of DEC in the UV range of 200–900 nm. DEC showed a maximum absorption peak at 330 nm .Hence, this wavelength was used to conduct further experiments. After the incubation period (2 h), the percentage of free DEC in the supernatant was calculated using the formula mentioned in materials and methods section, the EE % and DLC% were found approximately 91% and 49%, respectively.The potential interaction mechanism between DEC and ENG is illustrated in (Fig. 4).The ENG binds with DEC by non-colavent interaction. At step A there occurs a reaction between Elastin and crosslinker BS3 which results in the formation of ENG via amide bond.At step the drug Decursin is being incorporated and binds with ENG via end-end hydrogen bonding, facilitates encapsulation of DEC within the ENG^28^.

### Nuclear magnetic resonance (NMR) analysis

**Figure 5:**
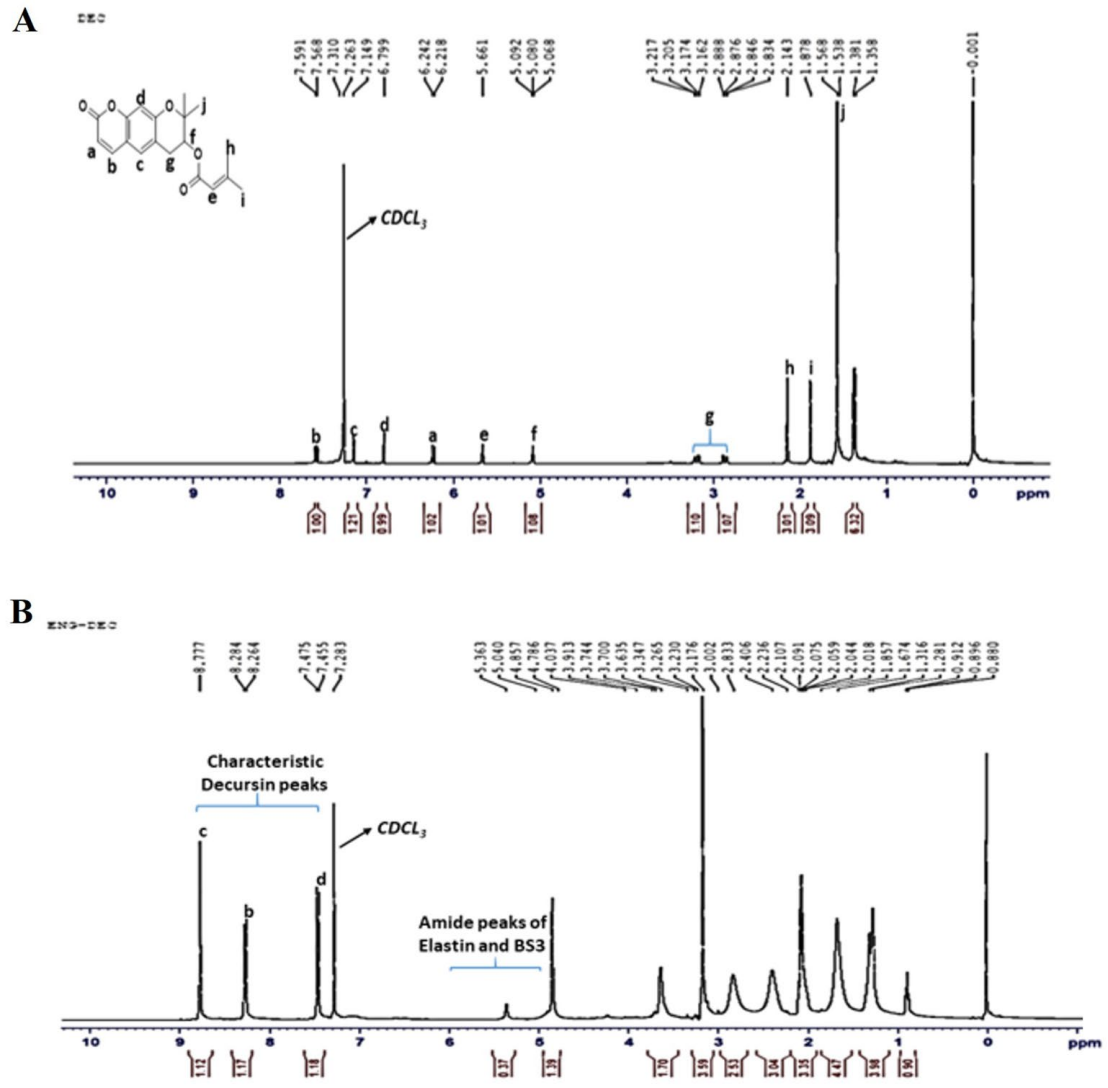
^1^HNMR studies of the ENG and decursin (DEC). (**A**) ^1^H NMR of DEC and (**B**) ^1^H NMR of ENG-loaded DEC.

The presence of Decursin drug inside the ENG was confirmed through the proton nuclearmagnetic resonance spectroscopy (^1^H NMR). The NMRspectrum of the Decursin drug as shown in (Fig. 5A) exhibits singlet peaks at 1.56 ppm which corresponds to the methyl group protons. Peaks observed between 6–8 ppm correspond to the aromatic protons of the drug molecule. The NMR spectra of DEC incorporated ENG exhibits peaks that correspond to the drug molecule as well which clearly shows the presence of DEC drug inside the ENG. The drug is interacting with the ENG through the carbonyl oxygen of six membered ring with the NH of N-methylpyrrolidine of the Elastin, this was confirmed through proton nuclearmagnetic resonance spectroscopy (^1^H NMR) of DEC-loaded ENG as the peak intensity at 5.36 ppm corresponds to NH of N-methylpyrrolidine is getting decreased. The ^1^H NMR spectra of DEC-loaded ENG shows some other characteristics peaks from 7.28–8.77 ppm which corresponds to the aromatic benzene ring and one double bond in ring of DEC drug as ENG does not contain aromatic protons shown in (Fig. 5B) which clearly gives confirmation that drug got incorporated in ENG.

### FTIR analysis

**Figure 6:**
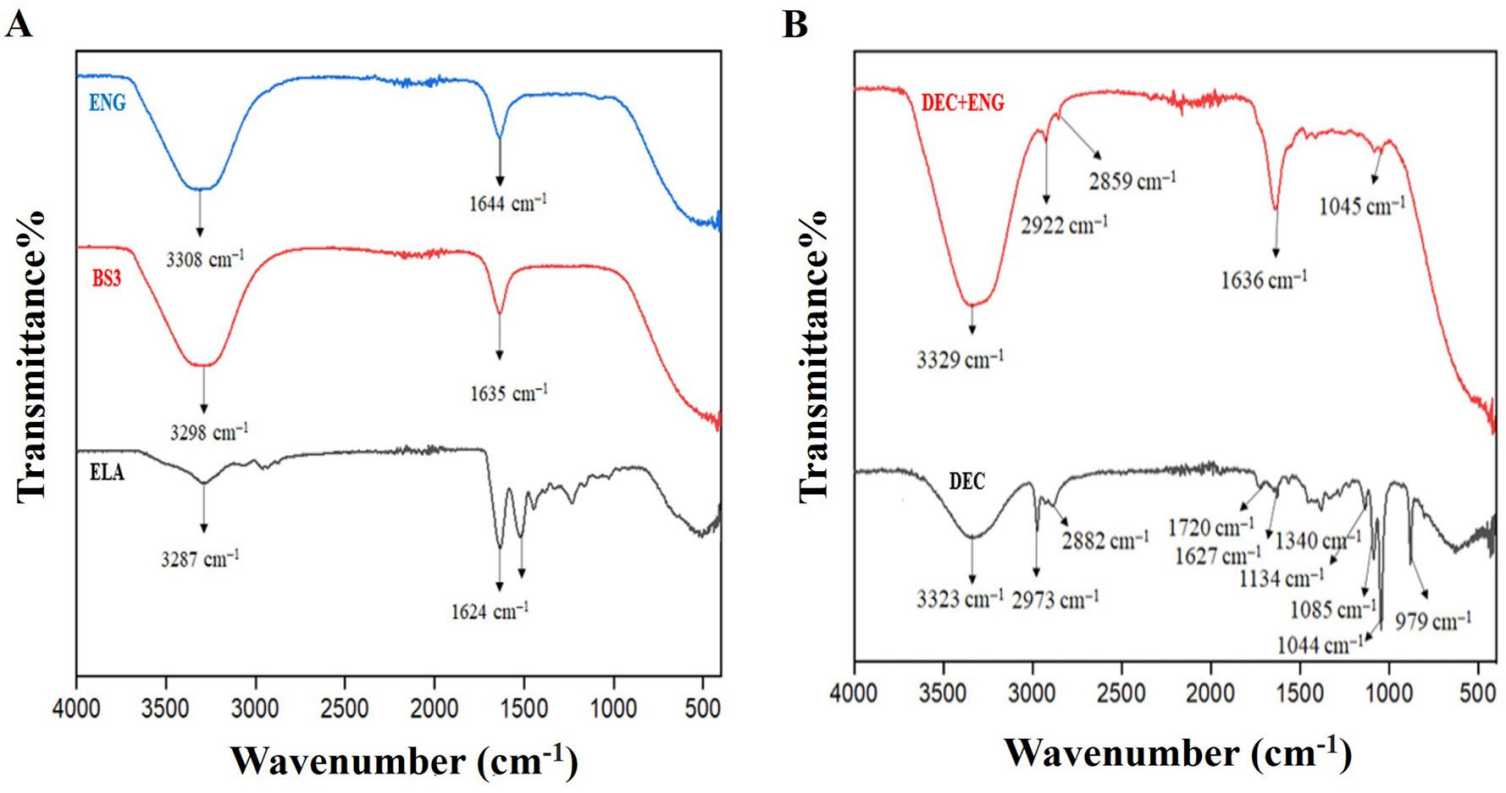
The Fourier-transform infrared (FTIR) spectra analysis of (**A**) ENG, BS3, and pure ELA and (**B**) DEC and DEC + ENG Abbreviations: *ENG* elastin nanogel, *BS3* bis (sulfosuccinimidyl) suberate, *ELA* elastin, *DEC* decursin.

The FTIR spectra of ENG, BS3, ELA, DEC + ENG, and DEC are depicted in (Fig. 6A,B),The peaks present at 1644, 1635, 1624, 1636, and 1627 cm^−1^ correspond to amide bond I (C = O stretching) in the spectra of ENG, BS3, ELA, DEC, and DEC + ENG ^19^.The peaks present at 2882, 2973, 2859, and 2922 cm^−1^ in the spectra of DEC and DEC + ENG correspond to aliphatic or saturated hydrocarbons and methyl (CH3) or methylene (CH2) groups. Moreover, these peaks correspond to C-H stretching^29^.The peaks present at 3308, 3323, and 3329 cm^−1^ in the spectra of ENG, DEC, and DEC + ENG correspond to the stretching vibrations of N–H bonds. The peaks present at 3287 and 3298 cm^−1^ in the spectra of ELA and BS3 correspond to -OH stretching vibrations^30^. Comparing the free drug (DEC) with drug-loaded ENG (DEC + ENG), shifts in the peaks from 1,085 to 1,045 cm^−1^ and from 1627 to 1636 cm^−1^ were observed; these shifts corresponded to the intermolecular hydrogen bonding between DEC and ENG. A shift in the peaks of DEC and DEC + ENG at 3308–3329 cm^−1^ indicated the interaction of DEC with ENG.

### In silico analysis to the DEC-AR interaction

**Figure 7:**
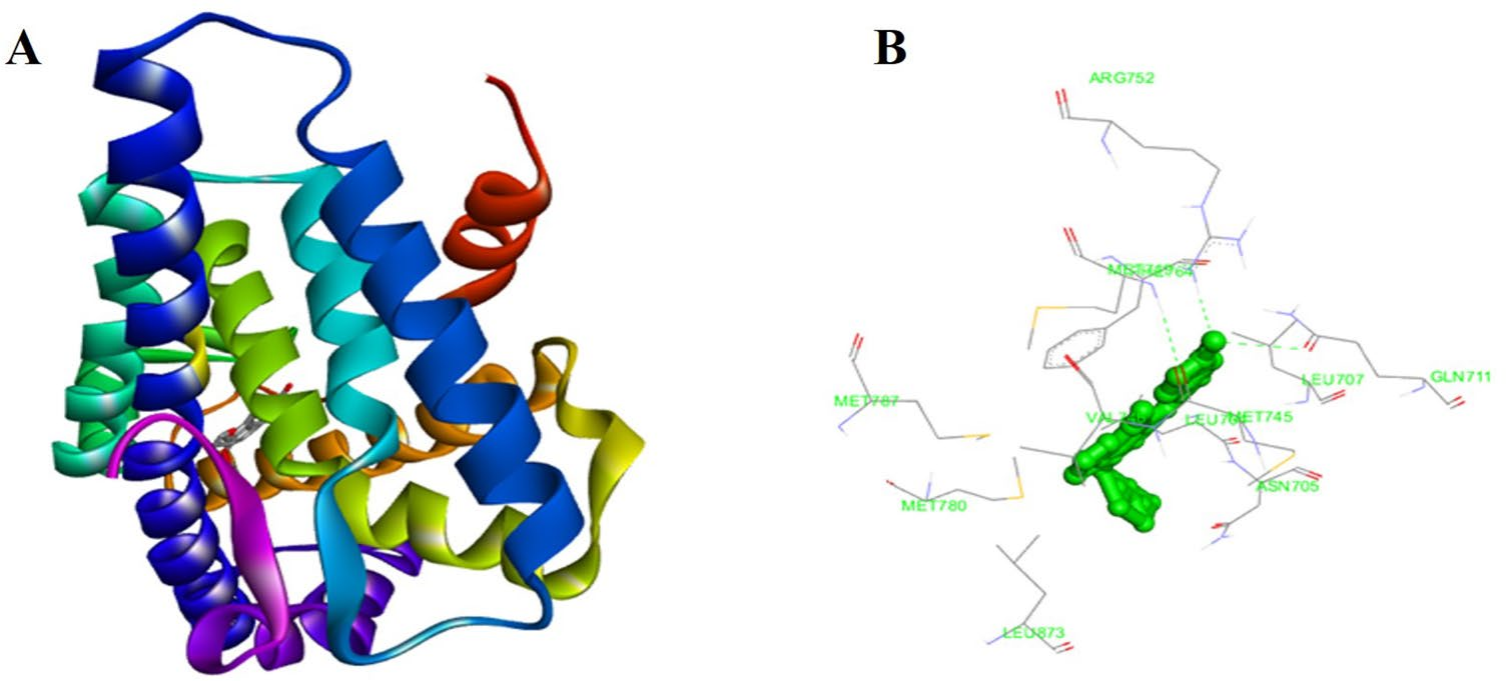
Molecular docking study. (**A**) 3D docking pose and (**B**) the binding interaction of decursin (green) with the human androgen receptor (blue).

The 3D docking pose and the binding interaction of DEC with AR are presented in (Fig. 7A, B). According to the docking results, DEC and AR demonstrated a binding score of -11.1 kcal/mol, indicating a strong interaction between them. This binding was primarily facilitated by hydrogen bonding and hydrophobic interactions.DEC exhibited three hydrogen bond interactions with AR firstly, involving the hydrogen atom form the AR GLN^711^ residue of AR and the oxygen atom of DEC (NH…O = 1.88 Å, dihedral angle = 156.40º), secondly with the hydrogen atom from the AR MET^745^ residue and the oxygen atom of DEC (NH··· = 1.63 Å, dihedral angle = 89.17º) and lastly with the hydrogen atom from the AR VAL^461^ residue and the oxygen atom of DEC (NH···O = 1.80 Å, dihedral angle = 160.53º).Besides the hydrogen bonding ,the stability of the docking models were further stabilized by some other hydrophobic interactions between DEC and the ARG ^752^, ASN ^705^, LEU ^704^, LEU^707^, LEU ^873^, MET ^746^, MET ^749^, MET ^780^, MET ^787^, and PHE ^764^ residues of AR.

### Cumulative drug release

**Figure 8:**
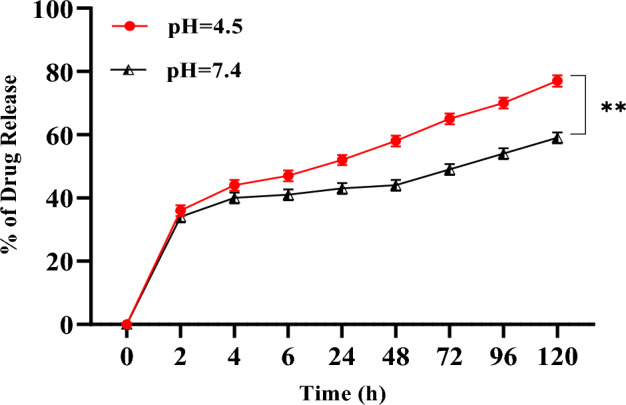
Release of decursin from the ENG at two different pH ranges. Data are presented as the mean ± SD (n = 3) **p < 0.05.

**Figure 9:**
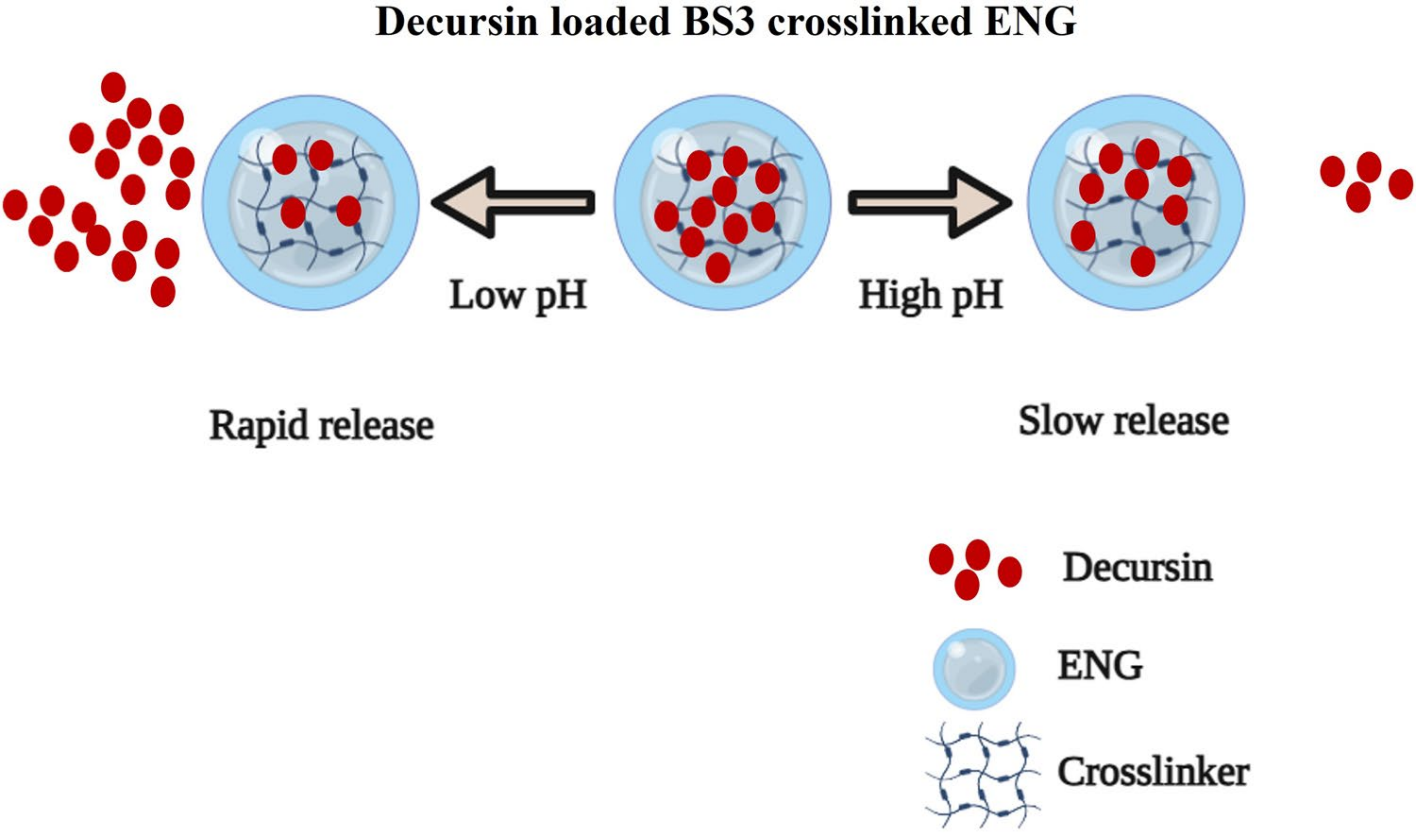
Shows the release of DEC from ENG in response to change in pH. (This image created using biorender.com).

The in vitro drug release of DEC was studied using PBS. DEC, as a model hydrophobic anticancer drug, was successfully loaded into the ENG. The in vitro drug release behaviour of DEC was evaluated under two different pH conditions using a dialysis membrane. The release of DEC from ENG was estimated using UV–Vis spectrophotometry (N60 Nanophometer, Implen, Munich, Germany) analysis at 330 nm. The drug release experiment showed that DEC exhibited faster release under acidic conditions (pH 4.5) than under neutral conditions (pH 7.4), as shown in (Fig. 8) and the difference in the release rates at two different pH values indicated that ENG responded mostly to acidic pH. DEC showed faster release in acidic conditions owing to the weakening of the binding between DEC and ENG, which improved DEC solubility at low pH^31,32^. This pH-dependent drug release behaviour forms the basis for the use of DEC-loaded ENG in targeted drug delivery for PCa therapy. The equilibrium between swelling and de-swelling in nanogels is predominantly governed by variations in pH, resulting from the ionization of amide groups. Consequently, under acidic conditions, the increased flexibility and swelling of elastin lead to a greater release of Decursin. Several authors have mentioned this phenomenon for stimulus responsive nanogels^33^. Under acidic conditions the pore size of the ENG increases due to swelling which results in the faster release of DEC (Fig. 9). Moreover, at low pH the strength of the intramolecular hydrogen bonding loosening between DEC and ENG, which results in faster release of DEC from the compared to high ph. The drug release curve shows the primary burst release at both the pH conditions which may be due to the release of surface un-bound DEC molecules, followed by cumulative release. The cumulative drug release at pH = 4.5 and pH = 7.4 was ~ 77% and ~ 59% respectively.

### In vitro cytotoxicity

**Figure 10:**
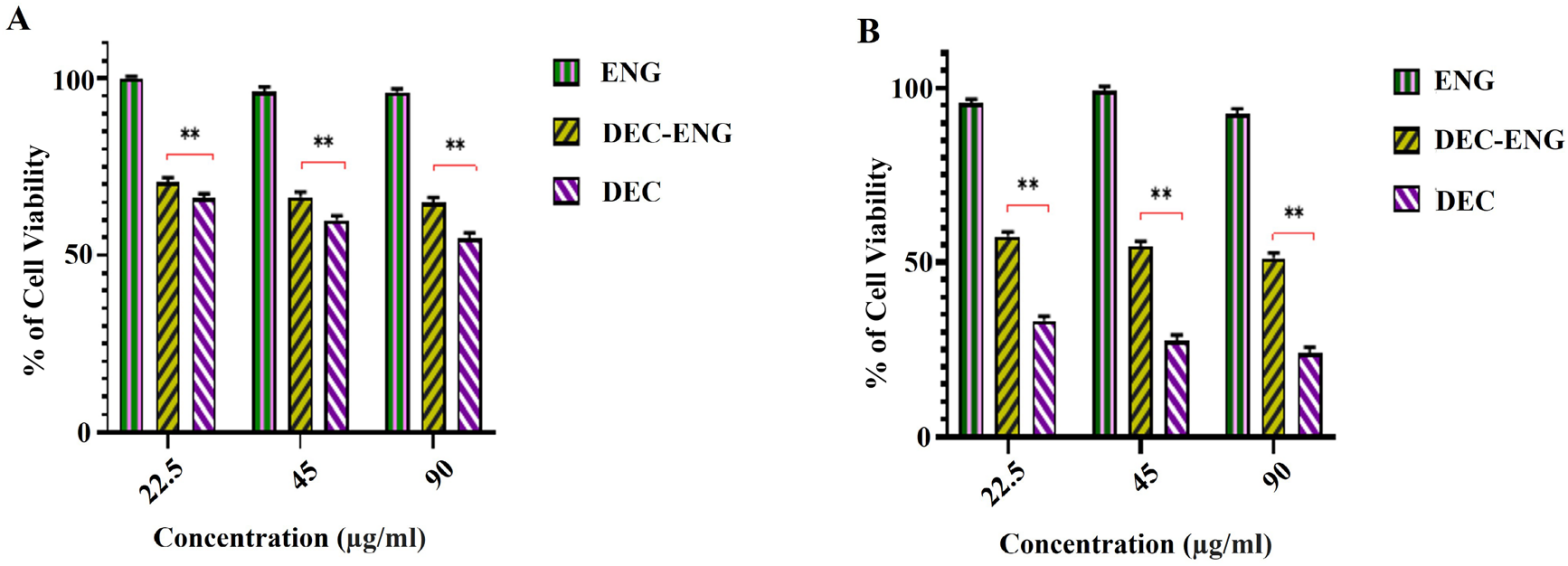
Cytotoxicity of the blank ENG, DEC-loaded ENG and free DEC, as determined the neutral red assay with DU145 cells for **(A)** 24 and **(B)** 48 h. Data presented as the mean ± SD (n = 3).**p < 0.05.

To determine, the cytotoxicity of pH-responsive ENG, cytotoxicity evaluations were conducted using the neutral red assay (NRA). In this study, the NRA of blank-ENG, DEC-loaded ENG, and free DEC was carried out at 24 and 48 h, as depicted in (Fig. 10 A, B).The blank nanogel did not show toxicity clearly shows that free DEC causes more toxicity to DU145 cells than DEC-loaded ENG, and this effects exhibits a dose dependent relationship. The pH-responsive ENG releases encapsulated DEC in a slightly acidic tumor extracellular microenvironment and prolongs the anticancer activity of DEC^28^. The cytotoxicity of elastin nanogel loaded with decursin (DEC + ENG) is marginally less than that of free decursin (DEC). In a comparable investigation, a similar impact was observed between doxorubicin nanogel and free doxorubicin^34^.

### Morphological study of DU145-cells

**Figure 11:**
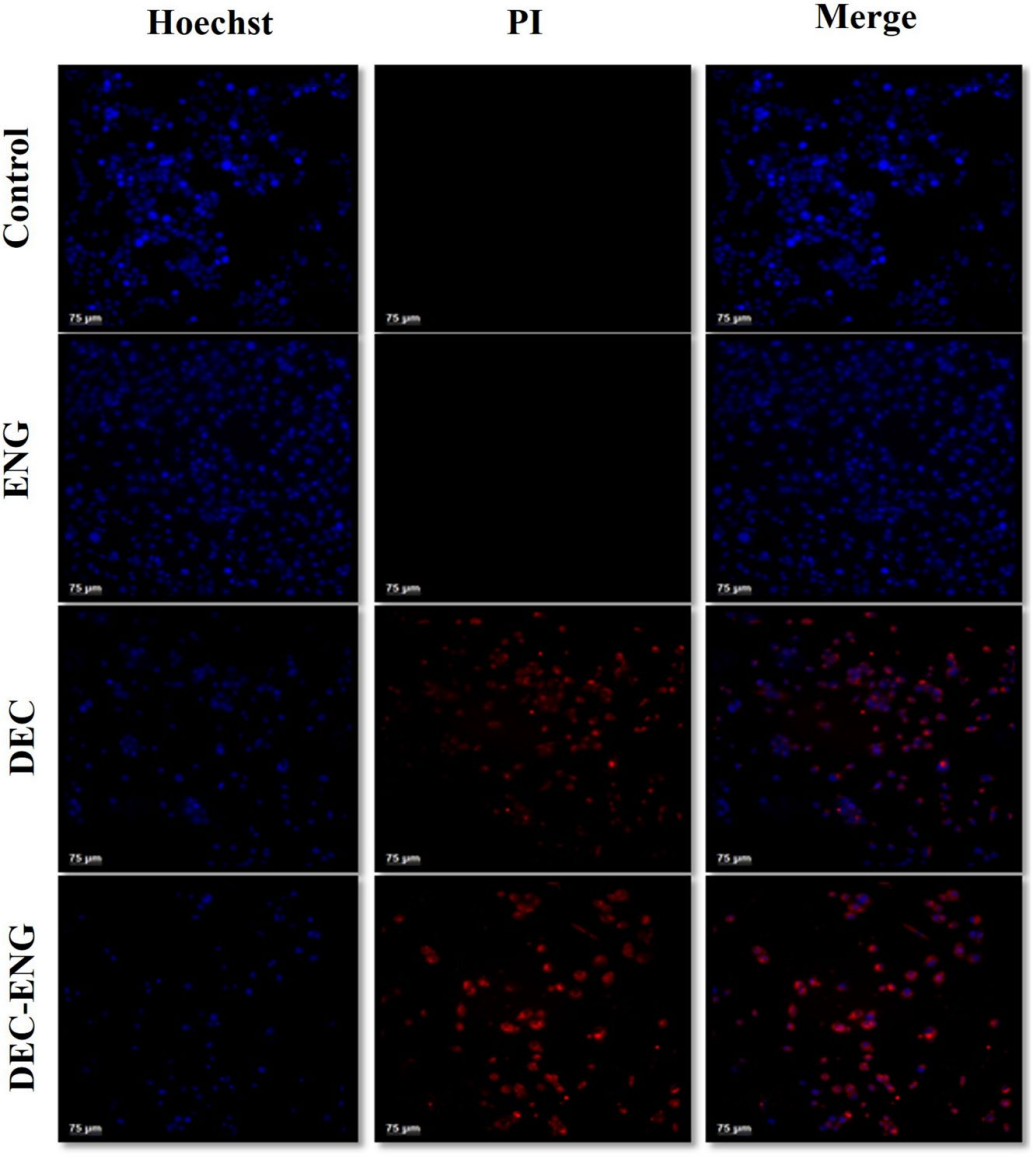
Illustrates the morphological examination of DU145 cells through Hoechst 33,342/PI staining using confocal microscopy. The first row depicts untreated cells (control), the second row shows cells treated with ENG, the third row displays cells treated with DEC, and the fourth row shows cells treated with DEC-ENG for 48 h.

Morphological alterations resulting from ENG, DEC, and DEC-ENG were evaluated through Hoechst 33,342/PI staining by confocal microscopy. In both the control and ENG groups, all cells exhibited a blue stain, with no presence of red cells. However, in the presence of the drug (DEC) and drug-loaded Nanogel (DEC-ENG), an increased occurrence of red-stained cells and a reduction in cell count were observed, indicating the induction of cell necrosis, cell shrinkage, nuclear elongation and cell death^35^ (Fig. 11) .The number of dead cells in drug (DEC) and drug-loaded Nanogel (DEC-ENG) are more indicating that ENG acts as an effective nano-carrier in DU145 cells.

## Conclusion

In this study, we have designed pH- responsive ENG for the delivery of DEC for PCa therapy to address the usefulness of elastin nanogels compared to current routine use of DC beads in clinic. Our ENG demonstrated desirable properties, excellent encapsulation efficiency (EE %) around 91% and excellent particle size approximately 152.9 ± 0.968 nm suitable for biological applications. Furthermore, the in vitro drug release studies confirmed the sustained release of DEC from ENG. Notably, the rate of DEC release was higher at pH 4.5 than at pH 7.4, indicating the pH-dependent behaviour of the ENG. These findings suggest that the designed ENG can respond to changes in pH and temperature, offering controlled release and targeted therapy*. *In silico study demonstrated the strong affinity of DEC towards human AR. The in vitro cytotoxicity assessment revealed moderate effect underscores the potential of pH-responsive ENG to facilitate the controlled release of encapsulated DEC with Enhanced Permeability and Retention (EPR) effect. Our morphological analysis revealed uptake of ENG-DEC measureable suggesting platform for targeting cluster differentiation molecules such as CD24 and thereby enhancement of chemo sensitization of enzalutamide drug especially for CRPC.

## Materials and methods

Elastin powder extracted from bovine neck ligament were procured from Sigma Aldrich (St. Louis, Mo, USA), crosslinker Bis-sulfosuccinimidyl suberate (BS3), molecular weight (MW) = 572.43 g/mol) was obtained from Thermo Fischer Scientific (Waltham, MA,USA). Span-80, Tween-80, n-hexane (extra pure AR, 99%), tris acetate buffer, and dimethyl sulphoxide (DMSO AR, 99.5%) were purchased from SRL. DEC (HPLC-grade, ≥ 97%, MW = 328.36 g/mol) was purchased from Sigma-Aldrich. Dialysis membrane molecular weight cut-off (MWCO = 12,000–14,000) were purchased from Hi-Media (Mumbai, India). Prostate cancer cell line DU145 was purchased from National Centre for Cell Sciences (NCCS), Pune, India. Hoechst 33,342 PI Dual staining assay kit (Cat #220,011) was obtained from RealGeneLabs (Ghaziabad, India).

### Preparation of ENG using the mini emulsion technique

Recently, we reported the preparation of ENG^12^. In the present study, ENG was prepared similarly, but with slight modifications, using the inverse mini-emulsion technique. In the continuous phase, 4 mL of n-hexane was mixed with a 3:1 ratio of Span-80 and Tween-80. The aqueous phase containing 100 mg of elastin powder was dissolved in 250 µL of Tris (pH = 8.8). The aqueous phase was emulsified in a dropwise manner within the continuous phase, vigorously stirred using a magnetic stirrer for 15 min, and subsequently sonicated with a probe sonicator (Sonics Vibra-cell Model-CV18) at 5% of amplitude for 5 min under ice cold conditions. Bis (sulfosuccinimidyl) suberate (BS3) (2 mM) was added to the resultant solution to promote cross-linking, and the dispersion was further sonicated for 2 min. Subsequently, the solution was allowed to react under continuous stirring for 50 min. Next, 50 mM Tris was added to quench the unreached BS3. Following this, the obtained solution was centrifuged at 10,000 rpm for 30 min at 4ºC the supernatant was discarded. The pellet was washed once with n-hexane and thrice with phosphate-buffered saline (PBS) and Milli-Q water to remove the unbound elastin and surfactants. The obtained ENG was purified by dialysis membrane and stored at 4 °C for further analysis.

### Characterization of ENG

Various methods were utilised for the characterization of ENG. The surface morphology of ENG was examined by using Scanning electron microscopy by using FEI-Quanta FEG 200F. The Fourier-transform infrared (FTIR) spectrum data of all samples, including control elastin, ENG, and drug-loaded ENG were recorded using a Nicolet iS50 FTIR Spectrometer (Thermo Fischer Scientific). The size distribution of the ENG was measured using DLS (Nanotrac wave II; Microtrac Inc., Montgomeryville, PA, USA). The zeta potential of the ENG was recorded at 25 °C using a Malvern Pananalytical instrument (serial no. MAL1278106; Malvern Panalytical Ltd., Malvern, UK).

### Nuclear magnetic resonance (NMR)

The (^1^HNMR) spectra of DEC-ENG and free-DEC was determined by using a Bruker Avance 400 MHz NMR spectrometer (Bruker Corporation, Billerica, MA, USA). Chemical shifts are reported in delta (δ) units and part per million (ppm). Coupling constants are reported in Hertz (Hz), and 2 mg of DEC was dissolved in chloroform (CDCl_3_), with tetramethylsilane (TMS) as the internal standard.

### Swelling study

Three different pH conditions-acidic (pH = 4), neutral (pH = 7), and basic (pH = 9) were used to examine the swelling behaviour of ENG. The dried weight of nanogel was noted as Wo. Subsequently, the nanogel was dipped into the corresponding pH solution for 10 min. Then, the solution of the gel was removed and the weight of the wet gel was recorded as Ww. The experiment was carried out in triplicates. The swelling ratio was calculated using the following formula:

$${\text{Swelling}}\,{\text{ratio = }}\frac{{\text{Ww - Wo}}}{{{\text{W0}}}}$$

### Molecular docking studies

The evaluation of interaction affinities between DEC and the human androgenic receptor (AR) (PDB:1E3G) was conducted using Auto Dock Tool (ADT) version 1.5.6 docking program from the (Scripps Research Institute in La Jolla, CA, USA. The structure of DEC was initially created using Chem Sketch (Advanced Chemistry Development, Inc. (ACD/Labs), Toronto, ON Canada, www.acdlabs.com) and converted into pdb format using Open Babel (http://openbabel.org). Subsequently, the AR protein sequence (pdb format) was obtained from Protein Data Bank (http://www.rcsb.org/pdb). The receptor (1EG3) and drug (DEC) files were prepared using Auto Dock Tools. To prepare the receptor molecule. All water molecules were removed. Subsequently polar hydrogen atoms and Kollman charges were added to the receptor molecule. Later on, the other bonds were permitted to rotate and the bonds which are rotatable were assigned to ligand molecules. The AR molecule was confined within a three-dimensional box containing 30 points in each dimension (30 × 30 × 30 in X × Y × Z dimensions), with a fixed space of 0.372 Å. For docking calculations, the Lamarckian genetic algorithm was implemented into Auto Dock and run with default settings for the docking procedure. In 3D view the resulting docked positions were visualised by using the Biovia Discovery Studio Client (Dassault Systems, Velizy-Villacoublay, France), on the other hand LIG-PLOT plus (European Bioinformatics Institute, Cambridge, UK), was employed to visualise the hydrophobic interactions and hydrogen bonding in 2D.

### Drug encapsulation

ENG was loaded with DEC using the incubation method. DEC was added to the elastin solution at a concentration of 1 mg/mL. The solution was gently stirred at 700 rpm and incubated for 2 h and then centrifuged at 12,000 rpm. The concentration of DEC in the supernatant was determined based on a standard curve (0–14 µg/ mL; *r* = 0. 9993) developed using UV–Vis spectrophotometry (N60 Nanophometer N60, Implen, Munich Germany) analysis at 330 nm. (Fig.S1a, b supplementary information) The encapsulation efficiency (EE %) and drug loading (DL %) was calculated by using the following formula:

$$EE\% = \frac{Total\,\,\,mass\,\,\,of\,\,drug - free\,\,drug}{{Total\,\,\,mass\,\,\,of\,\,drug}} \times 100$$

$$DL\% = \frac{Total\,\,amount\,\,of\,feeding\,Decur\sin - free\,Decur\sin \,}{{Weight\,of\,nanogel}} \times 100$$

### Drug release study

The release of DEC from ENG was carried out using dialysis membrane (MWCO = 12,000–14,000).At two distinct pH conditions, pH = 4.5 and pH = 7.5 the DEC-loaded ENG (1 ml) were added in dialysis membrane and placed in buffer using PBS with dissolution volume of 50 mL at 37 ºC.At specific intervals (0,4,6,12,48,72,96 and 120 h) 1 mL of sample were with withdrawn from the buffer phase and, an equivalent volume of fresh buffer was added to maintain the dissolution volume. The concentration of the DEC was determined using UV–Vis spectroscopy (N60 Nanophotometer; *Implen,* Munich, Germany).The drug release concentration was calculated by using the following formula:

$${\text{Drug}}\,\,{\text{release}}(\% ) = \frac{{{\text{Amount}}\,\,{\text{of}}\,{\text{drug}}\,{\text{release}}\,\, \times \,100}}{{{\text{The}}\,{\text{Total}}\,{\text{amount}}\,{\text{of}}\,{\text{drug}}}}$$

### Cell viability evaluation using the neutral red assay

The cytotoxicity of blank ENG, decursin-loaded ENG, and free decursin was determined using the neutral red (3-amino-7-dimethylamino-2-methyl-phenazine hydrochloride) assay. The DU145 cells were seeded in 96 well plates at a density of (1 × 10^-4^cells per well) and incubated for 24 h. Different concentrations (22.5,45 and 90 µg/mL) of blank ENG, decursin-loaded ENG, and free decursin were added for 24 and 48 h.The stock solution (4 mg/mL)and working solution (10 µL neutral red/mL basal media) of neutral red stain were prepared and stored at 37 °C.After 24 and 48 h the media was removed and the cells were added to 100µL of neural red staining solution and incubated for 2 h at 37 °C. Following a 2 h incubation, the staining solution was removed and the cells were rinsed with PBS. Subsequently, 100 µl of de-staining solution containing (0.2 mL glacial aceteic + 10 mL water + 10 mL ethanol) were added into every well. The plates were agitated on a shaker for 10 min, and the absorbance was measured at 540 nm using the microplate reader. The cell viability was calculated by using the following formula:

$${\text{Cell}}\,{\text{viability}}(\% ) = ({\text{As/Ac}}) \times 100\%$$

where As and Ac denote the absorbances of the sample and control wells, respectively.

### Morphological observation

To analyse the morphological changes in DU145 cell line, Hoechst 33,342 and PI dyes were used. In order to observe the morphological changes, the DU145 cell line at the density 5 × 10^4^ cells were seeded and coverslips were placed in a 24 well plates and incubated overnight for cell adherence. Following the incubation time, the cells were treated with 90 µg/ml in 5% serum MEM and left for 48 h. After treatment, the coverslips were washed with PBS and staining were performed using 200μL stain (5μL of Hoechst 33,342 and 5μL of propidium iodide (PI) in 1 mL staining buffer). After adding the stain, the plates were incubated at 37 °C for 30 min and then stain was removed by washing the coverslips with PBS. The coverslips were removed from the well plates and placed upside down on a clean glass slide for imaging. Fluorescent imaging was performed using a Dmi8 Thunder microscope (Leica Microsystems, Germany).

### Statistical analysis

Graph Pad Prism (version9.0.0) was employed for carried out statistical analysis. The data was presented as the mean ± (n = 3) and a p value < 0.05 on a 2-tailed test was considered as significant.

## Supplementary Information

Supplementary Figure S1.

## Data Availability

Data is provided within the manuscript and supplementary information files.
