# Supplementary material for: Facile synthesis of elastin nanogels encapsulated decursin for castrated resistance prostate cancer therapy
Source: Sci Rep. 2024 Jul 2;14:15095. doi: 10.1038/s41598-024-65999-x (PMC11219748; doi:10.1038/s41598-024-65999-x)
Supplement: Supplementary file 1 — Supplementary Figure S1. [file 41598_2024_65999_MOESM1_ESM.docx]

**Supplementary data**

**Assessment of encapsulation efficacy and drug loading of DEC in ENG**

In the experimental studies, the λmax of DEC was found at 330 nm, which is the actual peak of DEC.Based on the λmax the calibration curve of DEC was plotted. A linear relationship was observed between the absorbance and DEC concentration from (0-14 µg/mL); the regression equation of the calibration curve was y = 0.2365x-0.0213, as shown in **Figure S1A** from the working standard solution, 3 µL of sample and blank were used to check the maximum absorption peak of DEC in the UV range of 200–900 nm. DEC showed a maximum absorption peak at 330 nm **Figure S1B.** Hence, this wavelength was used to conduct further experiments. After the incubation period (2 h), the percentage of free DEC in the supernatant was calculated using the formula mentioned in the Materials and Methods section. Using this method, the EE % and DLC%.


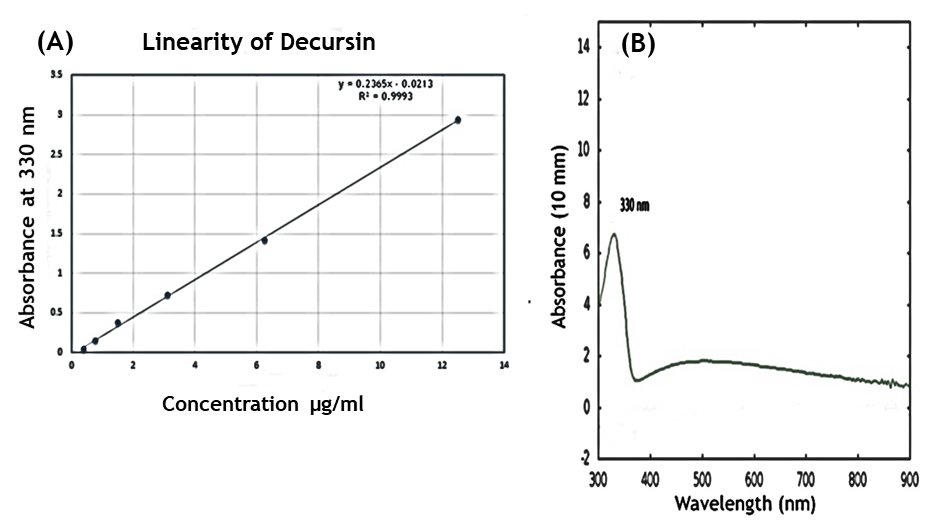


**Figure S1 The calibration curve of decursin (A) and UV–spectra analysis (B) of decursin.**
